# Trajectories of 24-Hour Physical Activity Distribution and Relationship with Dyslipidemia

**DOI:** 10.3390/nu15020328

**Published:** 2023-01-09

**Authors:** Xiaojing Wang, Yongjun Wang, Zechao Xu, Xiang Guo, Hongmei Mao, Tingting Liu, Weiyi Gong, Zhaolong Gong, Qin Zhuo

**Affiliations:** 1 Key Laboratory of Trace Element Nutrition of National Health Commission (NHC), National Institute for Nutrition and Health, Chinese Center for Disease Control and Prevention, Beijing 100050, China; 2 Department of Clinical Nutrition, The First Affiliated Hospital of Shandong First Medical University & Shandong Provincial Qianfoshan Hospital, Jinan 250014, China; 3 Beijing Chaoyang District Center for Disease Control and Prevention, Beijing 100050, China

**Keywords:** physical activity, temporal pattern, trajectory analysis, dyslipidemia

## Abstract

The association between physical activity (PA) and dyslipidemia is well known, but the relationship between a temporal pattern of PA and dyslipidemia remain unknown. Here, we aimed to identify the intensity and temporal patterns of PA clustered by the trajectory model and their relationship with dyslipidemia. The participants were 701 adults (305 males) aged 18–60 years undergoing continuous measurement of PA with Actigraph GT3X+ accelerometers for at least 3 days. A trajectory analysis was applied based on moderate-to-vigorous intensity physical activity (MVPA) accumulated values over every period per day. The association between PA and dyslipidemia was estimated using a logistic regression model. Four distinct PA trajectory groups in the population were identified (continued low, stable and moderate, late increasing, and early increasing). Specifically, the “moderate and stable group” was associated with a decreased rate of high TG (*p* < 0.05) and the “moderate and stable group” and “late increasing group” were associated with decreased rates of low HDL-C (*p* < 0.05). In conclusion, there were four activity trajectory groups in this population and the continued low PA trajectory was associated with a high prevalent rate of an abnormal lipid profile, and continued and moderate activity or late afternoon increasing activity might have lower HDL-C distribution.

## 1. Introduction

The health benefits of moderate-to-vigorous physical activity (MVPA) have been advocated for decades. Virtually all epidemiological studies have prompted advocating that people increase MVPA as an important part of preventing disease and premature mortality [1,2,3,4,5]. The effects of different types of MVPA-related exposure indicators and activity patterns are required to provide targets for physical activity (PA) intervention [6]. However, most researchers have focused on PA indicators collected by questionnaires. Benoit Salanave et al. [7] and Hsin-Yu An et al. [8] categorized the PA level as either high, moderate, or low based on the total metabolic equivalent analyzed by the International Physical Activity Questionnaire. This kind of evaluation is rough and subjective, neglecting plenty of PA information, such as the daily rest–activity patterns. Humans are a diurnal species and there are increasing studies dedicated to the search for the relationship between circadian rhythm and health. Circadian disruption has an effect on a wide range of preclinical and clinical disorders, including obesity and dyslipidemia [9]. Although researchers have not found a clear answer about the circadian regulation of lipid metabolism, it is well known that lipid pathways are under circadian control in all of the major metabolic organs [10]. Several studies have demonstrated that skeletal muscle and human body metabolism also have a strong circadian profile [11]. Saar Ezagouri et al., found that exercise performed at different times of the day influences skeletal muscle metabolic pathways and endurance capacity [12]. However, the relationship between the 24-h PA pattern and the health-related benefits is controversial. Few PA categories have been assessed considering both intensity-related and temporal MVPA distribution during the day.

Dyslipidemia is a prevalent risk factor for cardiovascular disease. Evidence from numerous studies has demonstrated that the modification of PA can favorably affect the total cholesterol (TC), high-density lipoprotein cholesterol (HDL-C), low-density lipoprotein cholesterol (LDL-C), and triglycerides (TG) [13,14]. However, there are few studies that have examined the relationship between circadian PA rhythms and dyslipidemia, especially based on objectively measured PA. The group-based trajectory model (GBTM) was developed to determine the subgroups within a given population based on various development trends in health-related indicators over time [15]. Although the trajectory model is traditionally used in cohort data, individual hourly MVPA time repeated measurements captured using a GT3X-accelerometer over 24 h is more similar for longitudinal data. Therefore, the application of the trajectory model to cluster underlying physical activity patterns in the population is likely to provide new insights and ideas into the development of a physical activity strategy and the prevention of metabolic diseases.

We therefore aimed to (1) cluster and characterize the 24-h physical activity pattern in a sample of 18–60 years adults and (2) to evaluate the health benefits of the clustered subgroups identified by examining the associations with dyslipidemia.

## 2. Materials and Methods

### 2.1. Study Participants

The analytic sample was drawn from a sample of 800 adults recruited from four provinces in China. The inclusion criteria were 18–60-year old adults. The exclusion criteria were a history disability that restricted physical activity; current or planned medication usage, or prior serious injuries/surgeries that influence physical activity; and women who were pregnant or breastfeeding. Ninety-nine participants were excluded from the present analysis because of incomplete accelerometry data, hemolytic blood samples, or incomplete dietary data. The final sample for the present analysis included 701 participants aged 18–60 years.

### 2.2. Accelerometer Data and Physical Activity Patterns

Participants wore an ActiGraph WGT3X-BT accelerometer (ActiGraph LLC, Fort Walton Beach, FL, USA) on their right hip continuously for at least three consecutive days, including two weekdays and one weekend. The accelerometer was initialized by a trained investigator with ActiLife V6.0 (ActiGraph R&D and Software Departments) data analysis software and was programmed to record the acceleration data acquired from the vector magnitude counts. Freedson (1998) bout parameters were used as the cut points to determine the intensity of activity (counts ≥ 2020 was determined to be MVPA). We screened the phenotypes of the accelerometer recorder expressed in the distribution of VM counts over 24 h, which was shown in ActiLife 6 software. According to the phenotypes of the accelerometer recorder and the lifestyles of most people, seven periods were divided from a 24-h period (03:00–06:00; 06:00–09:00; 09:00–12:00; 12:00–15:00; 15:00–18:00; 18:00–21:00; 21:00–03:00). The acceleration measurements of the MVPA time for each period were summed as the tracking data applied to the group-based trajectory modeling (GBTM) for the physical activity patterns.

### 2.3. Outcome Measures and Definition

Participants were required to maintain an empty stomach for 10–14 h before blood was drawn. The overnight fasting blood samples were collected by trained nurses and were stored in vacuum collection tubes. The samples were centrifuged at 3000 r/min for 10 min and the serum samples were taken, transferred to the frozen storage tube, and stored at −80 °C. The serum TC, TG, LDL-C, and HDL-C were measured by a HITACHI Automatic Analyzer (Japan, 7600-210) and Wako kit (Japan).

High TC was defined as TC ≥ 5.2 mmol/L and high TG was defined as TG ≥ 1.7 mmol/L. High LDL-C was defined as LDL-C ≥ 3.4 mmol/L and low HDL-C was defined as HDL-C ≤ 1.0 mmol/L. These were defined as borderline elevated blood lipids, according to the 2016 Chinese guideline for the management of dyslipidemia in adults [16].

### 2.4. Assessment of Covariates

The considered baseline variables that showed a univariate relationship with outcome without being on the causal pathway were entered into the multivariate regression model. The following measures were considered covariates: sex (male; female), age (<40 years; ≥40 years), job (non-manual worker (i.e., public official doctor student); manual worker (i.e., famer and builder)), BMI, and energy intake. In this study, the sex, age (<40 years; ≥40 years), job and education of participants were collected using a self-reported questionnaire; the BMI was measured by trained investigators; the energy intake data were collected based on three consecutive 24-h recalls combined with seasoning weighing, which was carefully explained in a previous study. Additional indicators of the participants were investigated: education (primary school; middle school; high school and above), total MVPA/day. An age of 40 years old was considered to categorize participants in the study because the prevalence of dyslipidemia among adults aged 40 years and over was high in the Chinese population [17,18].

### 2.5. Statistical Analysis

GBTM was used to identify the trajectory of the groups for the 24-h physical activity patterns, which defined latent groups in terms of both intensity and the distribution over time of the physical activity. The framework to construct and select the optimal trajectory groups depended on the model-adequacy criteria. Firstly, the data that met censored normal distribution were fitted into 1 to 5 trajectory groups, respectively. Secondly, the polynomial orders (linear, quadratic, and cubic) were tested in each of the trajectory groups. Finally, the optimal number of trajectory groups and the trajectory shape were determined based on the criteria of conciseness and model-adequacy, including the logged Bayes factor (≈2∆BIC, >10), average posterior probability of assignment (APPA, >0.70), and proportion of individuals estimated to be assigned to each group (≥1% for each group) [15]. This study empirically derived subgroups that had similar activity pattern characteristics using a censored normal modeling implemented with the package Proc Traj for SAS.

Baseline demographic, lifestyle, metabolism, and anthropometric variables were compared among the four trajectory groups. Analysis of variance was used for continuous variables with a normal distribution. The Kruskal–Wallis test and chi-square tests were used, where appropriate, for continuous variables with a non-normal distribution and for categorical variables, respectively. An association between the identified PA patterns and the prevalence of dyslipidemia was evaluated using logistic regression. Because of the design of this study, which is a cross-section study, odds ratio was used to evaluate the potency. To assess the effect of the covariates, three models were fitted. Model 1 was adjusted for no covariates. Model 2 was adjusted for age, sex, and job. Model 3 was additionally adjusted for BMI and total energy intake.

All of the analyses were conducted in SAS 9.4 (SAS Institute, Inc., Cary, NC, USA) and Graphpad Prism 9. *p* < 0.05 was considered to be statistically significant.

This study was reviewed and approved by the Ethics Committee of the Institute of Nutrition and Health of the Chinese Center for Disease Control and Prevention (no. 2019019). We confirmed that informed consent was obtained from all of the subjects.

## 3. Results

### 3.1. Identification of Latent Trajectory Groups for Physical Activity Patterns

**Figure 1:**
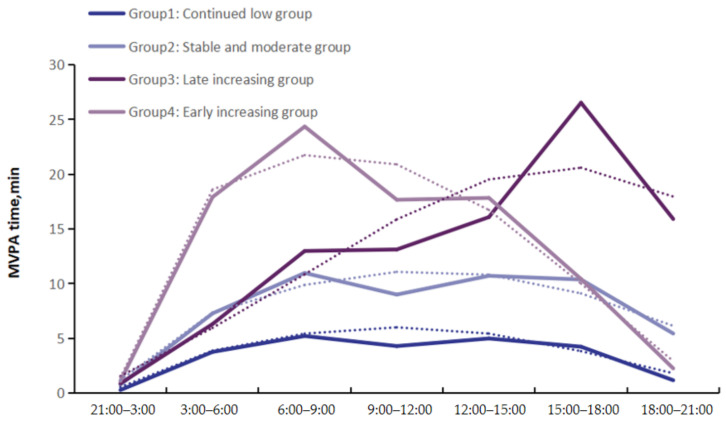
Trajectory groups of physical activity patterns in this study. Solid horizontal lines indicate the trajectory of the measured value for MVPA time (min). Dashed lines depict the predict trajectory for the four groups.

The cubic trajectory of four groups was clustered as the optimal trajectory model based on the model-adequacy criteria and the rule of concision, as shown in Figure 1. Table 1 shows the parameters of the model-adequacy criteria.

Group 1 was labeled as the “continued low group”, which comprised 35.1% of the participants, because the participants in this group were characterized by a low level of MVPA all day, and were not meeting the PA recommendation published by the WHO. Group 2 was the largest group, which comprised 53.7% of the participants, and was labeled as the “stable and moderate group”, because, on average, the participants in this group were characterized by the correct level of MVPA during daytime and had up to the recommended value for the average total MVPA. Group 3 was labeled as the “late increasing group”, and comprised 7.1% of participants, who were characterized by a high level of MVPA and a relatively higher physical activity in the afternoon. In contrast with Group 3, Group 4 was labeled the “early increasing group”, and comprised 4.0% of participants, who were characterized by a relatively higher physical activity in the morning and a sharp drop in the afternoon.

### 3.2. Characteristics of Participants in the Four Latent Trajectory Groups

The participants in Group 1 were often middle-aged and females with a high level of education, and most were non-manual workers. They likely had less energy intake and total MVPA time. The participants in Group 2 were often young and high level-educated females, and majority were non-manual workers. They had less energy intake. Group 3 were often young and males with a high level of education, and most were non-manual workers. They had the highest energy intake and total MVPA time. Group 4 were mostly higher-aged manual workers. They had a relatively high energy intake and total MVPA time, as shown in Table 2.

### 3.3. Association between the Physical Activity Trajectory Groups and Dyslipidemia

In this study, there was no significal difference for TC among the four trajectory groups, while there were significant differences for TG, LDL-C, and HDL-C (*p* < 0.001), as shown in Table 2. Associations between the four groups and the prevalence rates of dyslipidemia are shown in Table 3. Group 1 was identified as the reference group in this study for the previous evidence that less MVPA was related to dyslipidemia. Compared with Group 1, the “continued low group”, the odds of high TG and LDL-C and low HDL-C were lower in Group 2, the “stable and moderate group”, and Group 3, the “late increasing group”, without adjustment. There was no significant difference in the odds of high LDL-C (adjusted for age, sex, education, BMI, and energy intake) among the four groups. Compared with Group 1, the “continued low group”, the odds of abnormal TG (adjusted for age, sex, education, BMI, and energy intake) were lower in Group 2 (OR, 0.62; 95% CI, 0.39,0.99). Furthermore, compared with Group 1, the “continued low group”, Group 2 and Group 3 showed distinct protections for HDL-C (Group 2: OR, 0.25; 95% CI, 0.14,0.43; Group 3: OR, 0.09; 95% CI, 0.01,0.65). Overall, without adjustment, Group 2 and Group 3 showed low prevalence rates of high TG, high LDL-C, and low HDL-C compared with Group 1. The odds of high TG were lower in Group 2 and the odds of low HDL-C were lower in both Group 2 and Group 3.

## 4. Discussion

There were four potential physical activity patterns determined in the 18–60 year-old adults in this study: Group 1 was the continued low group, Group 2 was the stable and moderate group, Group 3 was the late increasing group, and Group 4 was the early increasing group. This study also observed that the four groups varied in demographic and anthropometric characteristics, as well as the prevalence of dyslipidemia. More importantly, compared with Group 1, the continued low group, the other three groups had a decreased risk of dyslipidemia after the adjustment for age, sex, and energy intake, and Group 3, the late increasing group, had the most protective effect. Compared with Group 1, the continued low group, Group 2 and Group 3 had decreased risks of high TG, high LDL-C, and low HDL-C without adjustment, and Group 3 had decreased risks of HDL-C after adjustment for age, sex, job, BMI, and energy intake.

Time of MVPA per hour in the 24-h period was identified as the tracking data to explore potential trajectory groups, taking into account both the cumulative time of MVPA and the circadian performance of PA in this study population. These analyses replicate and extend prior research in several ways. This study developed four groups: a low level of trajectory, a moderate level of trajectory, and other two trajectory with relatively high levels of PA, as well as a peak in the afternoon (15:00–21:00) and morning (06:00–12:00), respectively. Furthermore, this study found that one-third (about 35.6%) of participants followed a low level of physical activity all day, which is consistent with the worldwide prevalence rate of insufficient physical activity [19]. Half of participants (about 53.3%) followed a relatively moderate PA with an average of 55 min accumulated MVPA time per day, and most of them were young women with a relatively high education, while the majority of participants in Group 1 were the middle-aged population. These findings were consistent with previous studies that found that physical activity decreased with the addition of age [20,21]. Both Group 3 and Group 4 had high levels of physical activity, with a mean total MVPA time of 94 and 89 min per day, respectively. However, there was a peak at the tail end of the Group 3 trajectory, meaning that participants in this group likely did MVPA in the late afternoon, while participants in Group 4 were active in the morning. This study also found that participants in Group 3 were significantly younger than in Group 4, in line with previous findings that compared with younger adults (20–39 years), where older adults exhibited a reduced rest–activity rhythm amplitude, but reached their peak activity earlier [20,22].

A large number of studies have proven that human physical activity behaviors are affected by multiple factors due to social development [23]. Ecological models posit that the physical and social environments are important determinants of physical activity [24,25,26], for example, most physical activity is performed during leisure time [27] and thus farmers and elders do exercise in the morning, while college students and young people do exercise in the late afternoon. In addition, sedentary behavior is more common among middle-aged employees working in companies [28]. Therefore, there may indeed be different physical activity patterns associated with circadian rhythms in the population, and these findings have potential translational implications, in part, because accelerometers can export different kinds of metrics for 24-h physical activity, which can be used to explore physical activity patterns. As people have diverse lifestyles, identifying the underlying physical activity patterns in populations and exploring their associations with health outcomes can provide new insights to target in future mechanistic research and policy proposals.

In this study, the “continued low group” had the highest prevalence rate of high TC, TG, and LDL-C, and low HDL-C. Participants in this group spent an average of just 25 min on MVPA, below the WHO recommended physical activity level, while those in the other three groups reached the recommendation. These findings were complementary to previous studies, that physical inactivity can lead to dyslipidemia [29,30]. The “stable and moderate group” in this study had a low risk of high TG and low HDL-C, with either single factor analysis or multiple logistic analysis. Increasing studies have proven that PA is associated with a healthier blood lipid profile [31], and a randomized controlled trial showed that increased physical activity has a great effect on cholesterol biosynthesis, such as a decrease in TG level and an improvement in HDL-C level induced by regular PA [32,33]. Although the mechanisms underlying the effect of PA on the lipid profile are unclear, PA appears to enhance the ability of skeletal muscles to utilize lipids as opposed to glycogen, thus reducing the plasma lipid levels [34].

Both inactive and moderate activity levels have been found in most previous studies. Nevertheless, two groups with a relatively high activity level but a difference in the timing of activities were also present in this study population, and s relationship between the two groups and the lipid distribution profile was found. A lower risk of low HDL-C was associated with the “late increasing group”, while there was no significant protective effect for low HDL-C in the “early increasing group”. This finding suggests that more specific potential grouping involving time (morning and afternoon) in a population with similar levels of physical activity might be associated with lipid metabolism, and exercising in the late afternoon might be more beneficial for the HCL-C level than exercising in the morning. Similar to this finding, a randomized crossover trial suggested that late afternoon endurance exercise was more effective than morning exercise for at improving 24-h TG/HDL-C levels [35].

It is clear that skeletal muscle and cholesterol synthesis have strong circadian profiles [12,36], and exercise is a potent modulator of skeletal muscle metabolism [37]. The lowest rate of endogenous cholesterol synthesis being found during the day and the highest being found during the night means that the lipid profile may be more vulnerable for preventive measures at night, such as a more effective improvement in cholesterol levels following evening statin intake [38]. Moreover, a number of studies have demonstrated a higher activity metabolism rate in the evening than in the morning. However, the results of Yoshimi Fukuoka et al. [39] and Maisa Niemela et al. [40] were not in line with this study, as they found that HDL-C was lower in the evening active clusters compared with the moderately and very active clusters. This may be due to not specifying more detailed groupings and confounders, such as whether the early exercise group exercised before or after breakfast [41], and whether the late exercise group exercised in the late afternoon or at night. In this study, the “early increasing group” had a high level of activity in the morning, but a very low level in the late afternoon, which may also be a reason for its lack of protection against an abnormal lipid profile. Therefore, more detailed 24-h physical activity patterns and their combined effects need to be considered in future studies.

The strengths of the study include identifying time and intensity-related potential physical activity groups by using a trajectory model; the objective continuous measurement of physical activity; and the adjustment of age, sex, education, energy intake, and BMI as covariates to give a more reasonable relation between physical activity and dyslipidemia. Nevertheless, the study has some limitations. Firstly, accelerometer data were continuously measured for 3 days not 7 days. Although two weekdays and one weekend were measured for a better representative structure, it would be more stable and representative to have measured this for longer days. Secondly, although covariates were adjusted as much as possible, the possibility of other confounders unable to be included in this study could not be ruled out. Finally, it is notable that the scale of participants in this study was not sufficient to distribute enough people evenly across the four groups, and the cross-sectional data could not prove causation. Therefore, a larger population scale and age range with cohort investigation are needed for further study.

## 5. Conclusions

In this cross-sectional study, four temporal and intensity-related 24-h PA pattern groups were recognized by trajectory analysis in the free-living population in China and the characteristics differed among the four groups. A possible relationship between this pattern and dyslipidemia was suggested in this study. The “moderate and stable group” was associated with a decreased rate of high TG and the “moderate and stable group” and “late increasing group” were associated with decreased rates of low HDL-C. Further research is needed to investigate the biologic processes related to these behavioral phenotypes, including whether “late increasing” PA patterns improve the distribution of the lipid profile.

## Figures and Tables

**Table 1:** Model-adequacy criteria of the trajectory analysis.

Trajectory Group Number	The Logged Bayes Factor ^1^	APPA ^2^	Proportion of Individuals in Groups ^3^ (%)
1	NA	NA	1
2	208.96	0.89	59.5/40.5
3	41.36	0.89	62.9/32.5/4.6
4	66.58	0.85	35.1/53.7/7.1/4.0
5	26.46	0.84	35.7/52.4/7.3/3.4/1.1

^1^ The logged Bayes factor: ≈2∆BIC, >10; ^2^ APPA: average posterior probability of assignment, >0.70; ^3^ Proportion of individuals in groups: proportion of individuals estimated to be assigned to each group, ≥1% for each group.

**Table 2:** Characteristics of participants in the four physical activity trajectory groups.

Baseline Characteristics	Total	Group 1	Group 2	Group 3	Group 4	*p* Value
	N = 701 (100%)	N = 244 (35.6%)	N = 383 (53.3%)	N = 46 (7.1%)	N = 28 (4.0%)	
Age (%)						<0.001
<40 years	484 (69.0)	127 (52.1)	305 (79.6)	40 (87.0)	12 (42.9)	
≥40 years	217 (31.0)	117 (47.9)	78 (20.4)	6 (13.0)	16 (57.1)	
Sex (%)						0.028
Male	303 (43.2)	101 (41.1)	164 (42.7)	26 (55.3)	14 (50.0)	
Female	398 (56.8)	145 (58.9)	220 (57.3)	21 (44.7)	14 (50.0)	
Education (%)						<0.001
Primary school	121 (17.3)	27 (11.0)	79 (20.6)	9 (19.1)	6 (21.4)	
Middle school	162 (23.1)	72 (29.3)	72 (18.8)	5 (10.6)	14 (50.0)	
High school and above	418 (59.6)	147 (59.8)	233 (60.7)	33 (70.2)	8 (28.6)	
Job (%)						<0.001
non-manual worker	543 (77.5)	165 (67.1)	328 (85.4)	44 (93.6)	10 (35.7)	
manual worker	158 (22.5)	81 (32.9)	56 (14.6)	3 (6.4)	18 (64.3)	
Energy Intake (kcal/d, median (IQR))	1783.7 (737.5)	1699.3 (746.4)	1792.1 (683.5)	2015.5 (1045.6)	1949.8 (698.1)	<0.001
BMI (kg/m^2^, median [IQR])	22.0 (3.8)	22.2 (4.4)	21.9 (3.8)	22.0 (3.1)	22.7 (2.8)	0.135
MVPA (min/d, median (IQR))	46.4 (35.5)	23 (15)	55 (20)	94 (19)	89 (19)	<0.001
High TC (%)						0.090
Yes	181 (25.8)	72 (29.5)	94 (24.5)	9 (19.6)	6 (21.4)	
No	520 (74.2)	172 (70.5)	289 (75.5)	37 (80.4)	22 (78.6)	
High TG (%)						<0.001
Yes	122 (17.4)	66 (27.0)	49 (12.8)	3 (6.5)	4 (14.3)	
No	579 (82.6)	178 (73.0)	334 (87.2)	43 (93.5)	24 (85.7)	
High LDL-C (%)						<0.001
Yes	169 (24.1)	81 (33.2)	76 (19.8)	7 (15.2)	5 (17.9)	
No	532 (75.9)	163 (66.8)	307 (80.2)	39 (84.8)	23 (82.1)	
Low HDL-C (%)						<0.001
Yes	84 (12.0)	57 (23.4)	23 (6.0)	1 (2.2)	3 (10.7)	
No	617 (88.0)	187 (76.6)	360 (94.0)	45 (97.8)	25 (89.3)	

*p* < 0.05: there were significant differences among the four groups. Continuous variables with non-normal distribution were described by median and inter-quartile range (median (IQR)) and categorical variables were described by percentage of participants (%).

**Table 3:** Association between the physical activity trajectory groups and odds ratio of dyslipidemia.

Trajectory Groups	*n*	Model 1	Model 2	Model 3
		Odds Ratio (95% CI)	Odds Ratio (95% CI)	Odds Ratio (95% CI)
High TC				
Group 1	246	1	1	1
Group 2	383	0.77 (0.54,1.1)	1.09 (0.74,1.61)	1.10 (0.74,1.63)
Group 3	47	0.64 (0.30,1.36)	0.91 (0.40,2.04)	0.93 (0.41,2.11)
Group 4	28	0.67 (0.25,1.66)	0.54 (0.21,1.45)	0.57 (0.21,1.52)
High TG				
Group 1	246	1	1	1
Group 2	383	0.39 (0.26,0.60) *	0.59 (0.38,0.93) *	0.62 (0.39,0.99) *
Group 3	47	0.18 (0.06,0.61) *	0.31 (0.09,1.09)	0.38 (0.11,1.36)
Group 4	28	0.45 (0.15,1.33)	0.30 (0.09,0.93) *	0.35 (0.11,1.14)
High LDL-C				
Group 1	246	1	1	1
Group 2	383	0.49 (0.34,0.71) *	0.67 (0.45,1.00) *	0.71 (0.47,1.06)
Group 3	47	0.41 (0.18,0.92) *	0.50 (0.21,1.22)	0.58 (0.24,1.42)
Group 4	28	0.44 (0.16,1.19)	0.32 (0.11,0.92) *	0.38 (0.13,1.09)
Low HDL-C				
Group 1	246	1	1	1
Group 2	383	0.21 (0.12,0.35) *	0.24 (0.14,0.42) *	0.25 (0.14,0.43) *
Group 3	47	0.07 (0.01,0.52) *	0.07 (0.01,0.55) *	0.09 (0.01,0.65) *
Group 4	28	0.39 (0.11,1.34)	0.42 (0.12,1.52)	0.52 (0.14,1.85)

Model 1 adjusted for no covariates. Model 2 adjusted for age (categorical), sex (categorical), and job (categorical). Model 3 additionally adjusted for BMI and total energy intake. * *p* < 0.05.

## Data Availability

Data are available upon request due to ethical restrictions.

## References

[1] Young D.R., Haskell W.L. (2018). Accumulation of Moderate-to-Vigorous Physical Activity and All-Cause Mortality. J. Am. Heart Assoc..

[2] Farrahi V., Kangas M., Kiviniemi A., Puukka K., Korpelainen R., Jämsä T. (2021). Accumulation patterns of sedentary time and breaks and their association with cardiometabolic health markers in adults. Scand. J. Med. Sci. Sports.

[3] Gallardo-Alfaro L., Bibiloni M.D.M., Bouzas C., Mascaró C.M., Martínez-González M., Salas-Salvadó J., Corella D., Schröder H., Martínez J.A., Alonso-Gómez Á.M. (2021). Physical Activity and Metabolic Syndrome Severity among Older Adults at Cardiovascular Risk: 1-Year Trends. Nutr. Metab. Cardiovasc. Dis..

[4] Nakagawa T., Koan I., Chen C., Matsubara T., Hagiwara K., Lei H., Hirotsu M., Yamagata H., Nakagawa S. (2020). Regular Moderate- to Vigorous-Intensity Physical Activity Rather Than Walking Is Associated with Enhanced Cognitive Functions and Mental Health in Young Adults. Int. J. Environ. Res. Public Health.

[5] Cauley A.J., Giangregorio L. (2020). Physical activity and skeletal health in adults. Lancet Diabetes Endocrinol..

[6] Nagata J.M., Vittinghoff E., Gabriel K.P., Garber A.K., Moran A.E., Rana J.S., Reis J.P., Sidney S., Bibbins-Domingo K. (2021). Moderate-to-vigorous intensity physical activity from young adulthood to middle age and metabolic disease: A 30-year population-based cohort study. Br. J. Sports Med..

[7] Salanave B., Vernay M., Szego E., Malon A., Deschamps V., Hercberg S., Castetbon K. (2012). Physical Activity Patterns in the French 18-74-Year-Old Population: French Nutrition and Health Survey (Etude Nationale Nutrition Santé, Enns) 2006–2007. Public Health Nutr..

[8] An H.Y., Chen W., Wang C.W., Yang H.F., Huang W.T., Fan S.Y. (2020). The Relationships between Physical Activity and Life Satisfaction and Happiness among Young, Middle-Aged, and Older Adults. Int. J. Environ. Res. Public Health.

[9] Li J., Vungarala S., Somers V.K., Di J., Lopez-Jimenez F., Covassin N. (2022). Rest-Activity Rhythm Is Associated with Obesity Phenotypes: A Cross-Sectional Analysis. Front. Endocrinol..

[10] Gooley J.J. (2016). Circadian regulation of lipid metabolism. Proc. Nutr. Soc..

[11] Ezagouri S., Zwighaft Z., Sobel J., Baillieul S., Doutreleau S., Ladeuix B., Golik M., Verges S., Asher G. (2019). Physiological and Molecular Dissection of Daily Variance in Exercise Capacity. Cell Metab..

[12] Gutierrez-Monreal M.A., Harmsen J., Schrauwen P., Esser K.A. (2020). Ticking for Metabolic Health: The Skeletal-Muscle Clocks. Obesity.

[13] Arnett D.K., Blumenthal R.S., Albert M.A., Buroker A.B., Goldberger Z.D., Hahn E.J., Himmelfarb C.D., Khera A., Lloyd-Jones D., McEvoy J.W. (2019). 2019 ACC/AHA Guideline on the Primary Prevention of Cardiovascular Disease: Executive Summary: A Report of the American College of Cardiology/American Heart Association Task Force on Clinical Practice Guidelines. Circulation.

[14] Knaeps S., De Baere S., Bourgois J., Mertens E., Charlier R., Lefevre J. (2018). Substituting Sedentary Time with Light and Moderate to Vigorous Physical Activity is Associated with Better Cardiometabolic Health. J. Phys. Act. Health.

[15] Lennon H., Kelly S., Sperrin M., Buchan I., Cross A.J., Leitzmann M., Cook M.B., Renehan A.G. (2018). Framework to Construct and Interpret Latent Class Trajectory Modelling. BMJ Open.

[16] (2016). 2016 Chinese Guideline for the Management of Dyslipidemia in Adults. Zhonghua Xin Xue Guan Bing Za Zhi.

[17] Zhang F.-L., Xing Y.-Q., Wu Y.-H., Liu H.-Y., Luo Y., Sun M.-S., Guo Z.-N., Yang Y. (2017). The prevalence, awareness, treatment, and control of dyslipidemia in northeast China: A population-based cross-sectional survey. Lipids Health Dis..

[18] Liu X., Yu S., Mao Z., Li Y., Zhang H., Yang K., Zhang H., Liu R., Qian X., Li L. (2018). Dyslipidemia prevalence, awareness, treatment, control, and risk factors in Chinese rural population: The Henan rural cohort study. Lipids Health Dis..

[19] Guthold R., Stevens G.A., Riley L.M., Bull F.C. (2018). Worldwide Trends in Insufficient Physical Activity from 2001 to 2016: A Pooled Analysis of 358 Population-Based Surveys with 1·9 Million Participants. Lancet Global Health.

[20] Smagula S.F., Zhang G., Gujral S., Covassin N., Li J., Taylor W.D., Reynolds C.F., Krafty R.T. (2022). Association of 24-Hour Activity Pattern Phenotypes with Depression Symptoms and Cognitive Performance in Aging. JAMA Psychiatry.

[21] Whiting S., Mendes R., Abu-Omar K., Gelius P., Crispo A., McColl K., Simmonds P., Fedkina N., Andreasyan D., Gahraman H. (2021). Physical inactivity in nine European and Central Asian countries: An analysis of national population-based survey results. Eur. J. Public Health.

[22] Li J., Somers V.K., Lopez-Jimenez F., Di J., Covassin N. (2021). Demographic Characteristics Associated with Circadian Rest-Activity Rhythm Patterns: A Cross-Sectional Study. Int. J. Behav. Nutr. Phys. Act..

[23] Seefeldt V., Malina R.M., Clark M.A. (2002). Factors Affecting Levels of Physical Activity in Adults. Sports Med..

[24] Bauman A.E., Reis R.S., Sallis J.F., Wells J.C., Loos R.J., Martin B.W. (2012). Correlates of Physical Activity: Why Are Some People Physically Active and Others Not?. Lancet.

[25] Oyeyemi A.L., Ishaku C.M., Oyekola J., Wakawa H.D., Lawan A., Yakubu S., Oyeyemi A.Y. (2016). Patterns and Associated Factors of Physical Activity among Adolescents in Nigeria. PLoS ONE.

[26] Malone S.K., Patterson F., Grunin L., Melkus G.D., Riegel B., Punjabi N., Yu G., Urbanek J., Crainiceanu C., Pack A. (2021). Habitual physical activity patterns in a nationally representative sample of U.S. adults. Transl. Behav. Med..

[27] García-Fernández J., González-López J.R., Vilches-Arenas Á., Lomas-Campos M.d.l.M. (2019). Determinants of Physical Activity Performed by Young Adults. Int. J. Environ. Res. Public Health.

[28] Hamilton K., Fraser E., Hannan T. (2019). Habit-based workplace physical activity intervention: A pilot study. Occup. Med..

[29] Katulanda P., Dissanayake H.A., De Silva S.D.N., Katulanda G.W., Liyanage I.K., Constantine G.R., Sheriff R., Matthews D.R. (2018). Prevalence, Patterns, and Associations of Dyslipidemia among Sri Lankan Adults-Sri Lanka Diabetes and Cardiovascular Study in 2005–2006. J. Clin. Lipidol..

[30] Booth F.W., Roberts C.K., Laye M.J. (2012). Lack of Exercise Is a Major Cause of Chronic Diseases. Compr. Physiol..

[31] Hyvärinen M., Juppi H.K., Taskinen S., Karppinen J.E., Karvinen S., Tammelin T.H., Kovanen V., Aukee P., Kujala U.M., Rantalainen T. (2022). Metabolic Health, Menopause, and Physical Activity-a 4-Year Follow-up Study. Int. J. Obes..

[32] Homer A.R., Fenemor S.P., Perry T.L., Rehrer N.J., Cameron C.M., Skeaff C.M., Peddie M.C. (2017). Regular Activity Breaks Combined with Physical Activity Improve Postprandial Plasma Triglyceride, Nonesterified Fatty Acid, and Insulin Responses in Healthy, Normal Weight Adults: A Randomized Crossover Trial. J. Clin. Lipidol..

[33] Sanllorente A., Soria-Florido M.T., Castañer O., Lassale C., Salas-Salvadó J., Martínez-González M., Subirana I., Ros E., Corella D., Estruch R. (2021). A Lifestyle Intervention with an Energy-Restricted Mediterranean Diet and Physical Activity Enhances Hdl Function: A Substudy of the Predimed-Plus Randomized Controlled Trial. Am. J. Clin. Nutr..

[34] Mann S., Beedie C., Jimenez A. (2014). Differential Effects of Aerobic Exercise, Resistance Training and Combined Exercise Modalities on Cholesterol and the Lipid Profile: Review, Synthesis and Recommendations. Sports Med..

[35] Kim H.K., Furuhashi S., Takahashi M., Chijiki H., Nanba T., Inami T., Radak Z., Sakamoto S., Shibata S. (2022). Late-Afternoon Endurance Exercise Is More Effective Than Morning Endurance Exercise at Improving 24-H Glucose and Blood Lipid Levels. Front. Endocrinol..

[36] Jones P., Schoeller D.A. (1990). Evidence for diurnal periodicity in human cholesterol synthesis. J. Lipid Res..

[37] Gabriel B., Zierath J.R. (2019). Circadian rhythms and exercise—Re-setting the clock in metabolic disease. Nat. Rev. Endocrinol..

[38] Saito Y., Yoshida S., Nakaya N., Hata Y., Goto Y. (1991). Comparison between Morning and Evening Doses of Simvastatin in Hyperlipidemic Subjects. A Double-Blind Comparative Study. Arterioscler. Thromb..

[39] Fukuoka Y., Zhou M., Vittinghoff E., Haskell W., Goldberg K., Aswani A., Norman G. (2018). Objectively Measured Baseline Physical Activity Patterns in Women in the mPED Trial: Cluster Analysis. JMIR Public Health Surveill..

[40] Niemelä M., Kangas M., Farrahi V., Kiviniemi A., Leinonen A.-M., Ahola R., Puukka K., Auvinen J., Korpelainen R., Jämsä T. (2019). Intensity and temporal patterns of physical activity and cardiovascular disease risk in midlife. Prev. Med..

[41] Iwayama K., Kurihara R., Nabekura Y., Kawabuchi R., Park I., Kobayashi M., Ogata H., Kayaba M., Satoh M., Tokuyama K. (2015). Exercise Increases 24-h Fat Oxidation Only When It Is Performed before Breakfast. eBioMedicine.

